# Severe Weather, Band Practice, Coal Trucks, and Other Real-World Experiences in Conducting Focus Group Research in Central Appalachia

**Published:** 2009-03-15

**Authors:** Robin C. Vanderpool

**Affiliations:** University of Kentucky College of Public Health, Department of Health Behavior

## Introduction

Despite our best intentions as methodical researchers who intricately describe their plans and processes in grant applications, we cannot anticipate everything that may occur when we conduct focus groups, particularly in a unique geographic region of the country. As project leader on a pilot project that addressed cancer survivorship issues in Appalachian Kentucky and Virginia (1), I learned that no textbook or mentor can prepare you for the experiences you have when conducting qualitative research in rural communities. This essay recounts several of the lessons our research team learned while we conducted focus groups with Appalachian cancer survivors and primary care providers from October 2007 through April 2008.

## Weather, Wildlife, and Travel Conditions

While weather influences traveling to and conducting research in any area, the mountainous topography of Appalachia requires special consideration. For example, we found that focus group participants may not show up for their sessions during thunderstorms because driving conditions deteriorate quickly on winding mountain roads. On one occasion, during a heavy downpour, we missed our exit and ended up on a poorly lit mountain parkway; we didn't even realize where we were until the road ended. The next day, the local news reported that there had been 2 tornados in the area.

Because it was winter, we expected snow in the mountains, but we were unprepared for the mixture of snow, sleet, and freezing rain that blanketed Lexington, where most of the researchers lived. Towns in the mountains, however, were not touched by the storm, and we felt obligated to hold the focus group because of participants' vested time and interest in the project. People who live in the mountains deal with this type of weather all winter long, and it doesn't hamper their daily activities, so we left earlier than planned and drove with extra caution.

**Figure 1.**
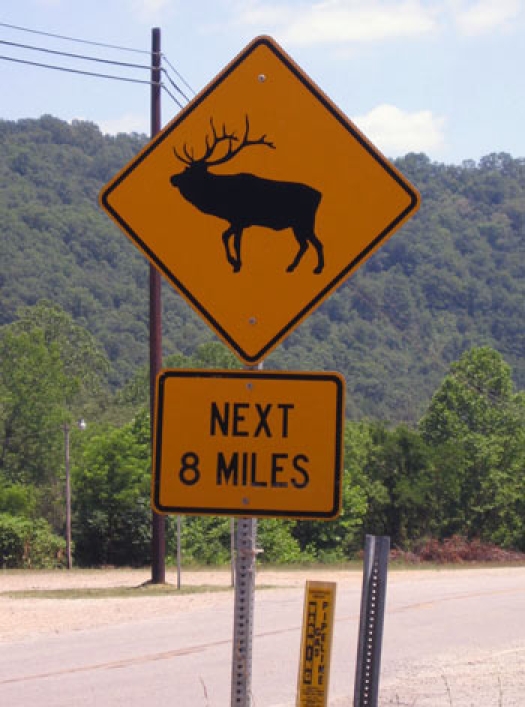
Bull elk roam wild in the Daniel Boone National Forest in Kentucky.

In the Daniel Boone National Forest in Appalachian Kentucky, bull elk have been reintroduced into the wild (2) (Figure 1). These animals are among the largest mammals in North America; they weigh an average of 700 pounds and stand an average of 5 feet high at the shoulder (3). Driving in this area, especially at night, is nerve-wracking because hitting an elk with a car can be a devastating experience for everyone involved. This circumstance is unique in this area; the signs — much less the animals themselves — are not seen anywhere else in Kentucky.

Once, as I was driving home late at night down an unlit, wet, curvy 2-lane highway that I was unfamiliar with, an animal with wolflike features greeted me by the shoulder of the road — right at the Wolfe County line, appropriately enough. I didn't want to hit the animal with my car or risk wrecking if I swerved to miss it. The possibility of being in a wreck in this area is frightening because cell phone service is spotty at best, and houses are few and far between. Fortunately, the suspected wolf was content to stay put and watch me drive by. The next day, the local county extension agent assured me that it was probably a male coyote, but he didn't dismiss the possibility that it could have been a wolf because wolves inhabit the area around the mountains.

Navigation software, such as MapQuest or Google Maps, is often not helpful in Appalachian Kentucky and Virginia. Many of the roads in this area do not exist on computer-generated maps. Fortunately, on the occasions that we got lost and needed to ask for directions, the residents of Appalachia were friendly and glad to point us in the right direction.

**Figure 2.**
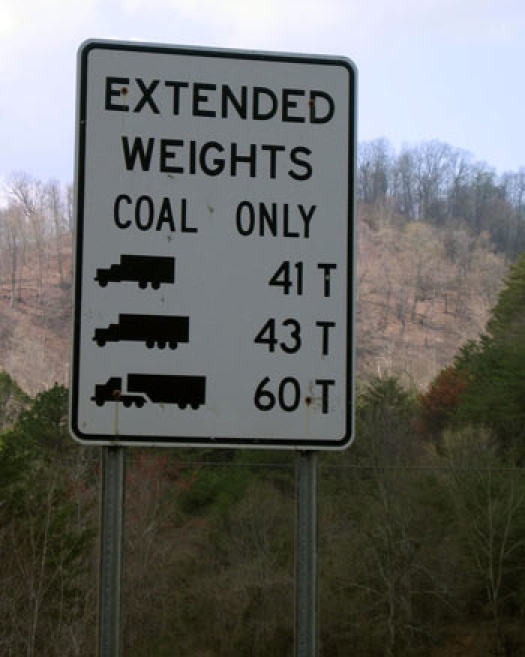
Coal trucks are ubiquitous on rural highways in Appalachia..

Researchers traveling to central Appalachia should be prepared for accidents and construction on interstates 64 and 75, among the busiest transportation corridors in the country and primary connectors to smaller mountain parkways and rural highways in Kentucky. Coal trucks are ubiquitous, and we are grateful to the Kentucky Department for Highways for designing truck lanes. Being stuck behind a 60-ton coal truck trundling slowly up a mountain on a rural 2-lane highway is a uniquely frustrating experience, as the residents of eastern Kentucky will attest (Figure 2). We quickly learned to budget extra time for traveling on these roads; if we estimated that a trip would take 2 hours, we allowed ourselves 4.

## Social Conditions

While conducting research in small, rural communities, such as those in central Appalachia, researchers must respect and learn from the local culture. Because of the close relationships among people in these areas, our community partners and their social networks were critical in planning focus groups and recruiting participants. "This is a family; this area is not just a community," observed one cancer survivor. The closeness of these small, rural communities was illustrated when a community member passed away, and our participants justifiably chose to attend the memorial service, instead of our focus groups.

Appalachian communities are extremely supportive of cancer survivors, particularly through faith-based networks. As a survivor explained, "I think faith is a very big part of this area. I know when I was going through it [cancer], there wasn't a single church that wasn't contacting me. I was on the prayer list of every church in the county; the Catholic, the Presbyterian, the Methodist, the Baptist, the Pentecostal, all these." Another survivor expressed a similar sentiment: "[The nurse] must have seen something in my face … and she said, 'Okay, let's just everybody get in a circle,' and she and the other oncology nurses gathered around and we prayed…. You wouldn't see that just anywhere…. We just joined hands and formed a circle, and we prayed in the middle of the hospital…. A lot of places might not, would maybe frown upon that."

At a regional Appalachian hospital where we held a primary care provider focus group, striking nurses, represented by the Kentucky and West Virginia Nurses Association Union/United American Nurses and supported by the United Mine Workers Association and United Steel Workers, protested along the busy road in front of the hospital, demanding better retirement and medical benefits and additional staffing (4,5). As public health professionals, our research team was empathetic to the nurses' cause, and we were uncomfortable entering the building. While we did not discuss the strike during the focus group, it was likely to have been distracting to the participants, many of whom had friends and family members who were striking. Supporters of the nurses would honk their horns while driving by, and the noise could be heard throughout the medical campus.

At another focus group that was held in the student center at a local liberal arts college, the pep band suddenly began practicing for their halftime performance at the men's basketball game — in the room directly above our session. Fortunately, the group of cancer survivors laughed it off and continued to share their stories (and the home basketball team ended up winning the game).

At 5 of our 7 focus group locations, space was provided to us at no charge and without any questions because our community partner vouched for us and our project. In another example of the close, intertwined relationships in these communities, we were able to reserve the college's student center because the administrative assistant at the college had worked with our community partner, and while our partner was receiving chemotherapy for colon cancer, the administrative assistant helped with the decorations for our partner's daughter's wedding.

In rural, tight-knit communities, word of mouth is a reliable focus group recruitment strategy for both patients and providers. When asked anecdotally why she decided to attend the focus group, one participant commented, "Because [the community partner] asked me to. She goes above and beyond for us…. She gives 150% of herself … and she's retired." This type of response further demonstrates how community relationships can improve participation in focus groups and enhance the quality of research.

## Conclusions

Our experiences in rural Appalachia may be similar to situations that other researchers may have encountered during projects in unique geographic locales. By documenting our accounts, we focus on the challenges of conducting research in communities where the research project is a low priority, respecting the local culture is imperative, community partners are essential, and topography and weather can interfere with the research. As in any pilot project conducted with focus groups, we must maintain a sense of professionalism and commitment to the research.

Focus group research involves more than the mundane processes of scheduling meeting rooms, ordering refreshments, photocopying consent forms, bringing incentives, and preparing an interview guide. A well-prepared investigator should carry a local map, be familiar with the wildlife in the area, pack an umbrella and a rain coat, bring a small token of appreciation to community partners, and always leave an hour early. Above all else, investigators should appreciate that visiting a new place and meeting new people is often inspirational and that focus group research is a labor of love.

## References

[1] Vanderpool R, Dignan M, Schoenberg N, Love M, Kudrimoti A Understanding the cancer information needs of Appalachian cancer survivors and providers. Poster presented at the 4th Biennial Cancer Survivorship Research Conference: Mapping the New Challenges.

[2] Kentucky Department of Fish and Wildlife Resources 2007 Elk restoration update and hunting information.

[3] Rocky Mountain Elk Foundation Fast facts about elk.

[4] Jafari S Appalachian nurses on strike. USA Today.

[5] Appalachian Regional Healthcare ARH registered nurses vote in favor of contract ending 3-month long strike.

